# Nephron-Sparing Surgery for Unilateral Non-Syndromic Wilms Tumor in a 9-Day-Old Neonate

**DOI:** 10.3390/children13070966

**Published:** 2026-07-22

**Authors:** Silvia Ceccanti, Francesco Morini, Loredana Amoroso, Gian Marco Andreoli, Gabriele Masselli, Denis A. Cozzi

**Affiliations:** 1 Pediatric Surgery Unit, Department Maternal Infantile and Urological Sciences, AOU Policlinico Umberto I, Sapienza University of Rome, Viale Regina Elena 324, 00161 Rome, Italy; silvia.ceccanti@uniroma1.it (S.C.); francesco.morini@uniroma1.it (F.M.); 2 Pediatric Oncology Unit, Department Maternal Infantile and Urological Sciences, AOU Policlinico Umberto I, Sapienza University of Rome, Viale Regina Elena 324, 00161 Rome, Italy; loredana.amoroso@uniroma1.it; 3 Pediatric Radiology Unit, Department of Radiological, Oncological, Pathological Anatomy Sciences, AOU Policlinico Umberto I, Sapienza University of Rome, Viale Regina Elena 324, 00161 Rome, Italy; gianmarco.andreoli@uniroma1.it; 4 Emergency Radiology Unit, Department of Radiological, Oncological, Pathological Anatomy Sciences, AOU Policlinico Umberto I, Sapienza University of Rome, Viale del Policlinico 155, 00161 Rome, Italy; gabriele.masselli@uniroma1.it

**Keywords:** neonate, nephron-sparing surgery, Wilms tumor, non-syndromic, radical nephrectomy

## Abstract

**Highlights:**

**What are the main findings?**

- Neonatal renal tumors are rare and biologically distinct from those in older children; congenital mesoblastic nephroma is the most common, while Wilms tumor (WT) accounts for about 20% of cases.
- Neonatal WT usually presents at low stage with favorable histology and excellent outcomes, and radical nephrectomy remains the standard treatment.

**What are the implications of the main findings?**

- Nephron-sparing surgery (NSS) can be a safe and effective option in carefully selected non-syndromic WT patients when complete resection with negative margins is achievable, particularly in neonates who may benefit from long-term renal preservation.
- Broader use of NSS for unilateral Wilms tumor may be appropriate when supported by careful patient selection, meticulous surgery, and multidisciplinary decision-making.

**Abstract:**

Neonatal renal tumors are uncommon and generally associated with an excellent prognosis. We report the youngest patient to date with unilateral non-syndromic Wilms tumor successfully treated with nephron-sparing surgery rather than radical nephrectomy, which is the current standard of care. The patient subsequently received risk-adapted chemotherapy. At 18 months of follow-up, he remains asymptomatic and with symmetric differential renal volumes, consistent with preserved bilateral renal function. This case suggests that nephron-sparing surgery, when technically feasible, may represent a safe and effective treatment option for carefully selected neonatal renal tumors, offering a functional advantage over radical nephrectomy without compromising oncologic safety.

## 1. Introduction

Neonatal renal tumors are rare and exhibit distinct clinical features compared with renal tumors diagnosed later in childhood [1]. Most of these tumors are benign, with congenital mesoblastic nephroma representing the most common entity. By contrast, Wilms tumor (WT), the most frequent renal malignancy in childhood, accounts for only about 20% of renal tumors in the first month of life [2]. Notably, neonatal WT is most often characterized by a low stage, favorable histology, and excellent outcomes.

While radical nephrectomy remains the gold-standard surgical approach for pediatric renal tumors, nephron-sparing surgery (NSS) is being used in selected cases, with indications in children more restricted than those applied in adult kidney cancer. In children, NSS is generally reserved for imperative indications such as bilateral disease or a solitary kidney, and for relative indications including conditions predisposing to renal insufficiency or to metachronous tumors [3]. However, NSS for unilateral WT in the context of a normal contralateral kidney remains largely investigational. Moreover, very limited information is currently available regarding the feasibility and oncological safety of NSS for neonatal WTs [4,5,6,7,8].

We report a case of a non-syndromic WT diagnosed at birth and successfully treated with primary NSS followed by risk-adapted chemotherapy.

## 2. Case Presentation

**Figure 1:**
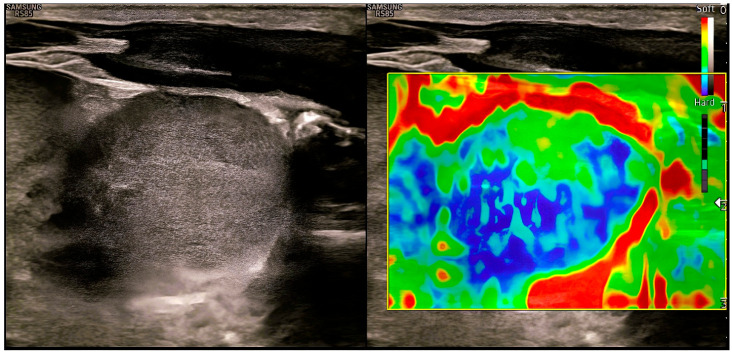
B-mode ultrasonography with elastography demonstrating a solid lesion arising from the lower pole of the left kidney. Color-coded elastography (**left**) reveals reduced elasticity (blue areas), indicating increased stiffness of the lesion and assisting in the characterization of pathological changes within the renal tissue.

**Figure 2:**
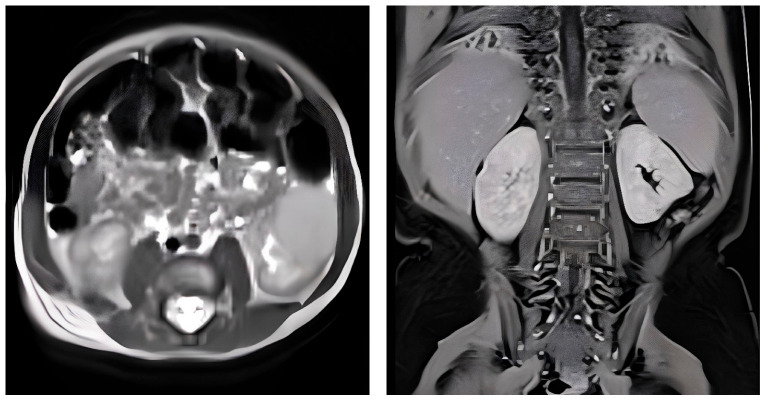
Pre- and post-operative MRI findings. (**Right**) Axial view of abdomen MRI at 3 days of life. Note a well-defined, homogenous mass occupying the lower anterolateral aspect of the left kidney. The lesion appears partially exophytic and cranially extends into the renal sinus. (**Left**) Coronal view of abdomen MRI at 8 months after surgery. MRI-based ellipsoid volume measurements showed that the operated kidney accounted for 44% of total renal volume. Given the close correlation between relative volume and function, this indicates preserved split renal function (conventionally defined as normal when each kidney contributes ≥40%) [11,12].

**Figure 3:**
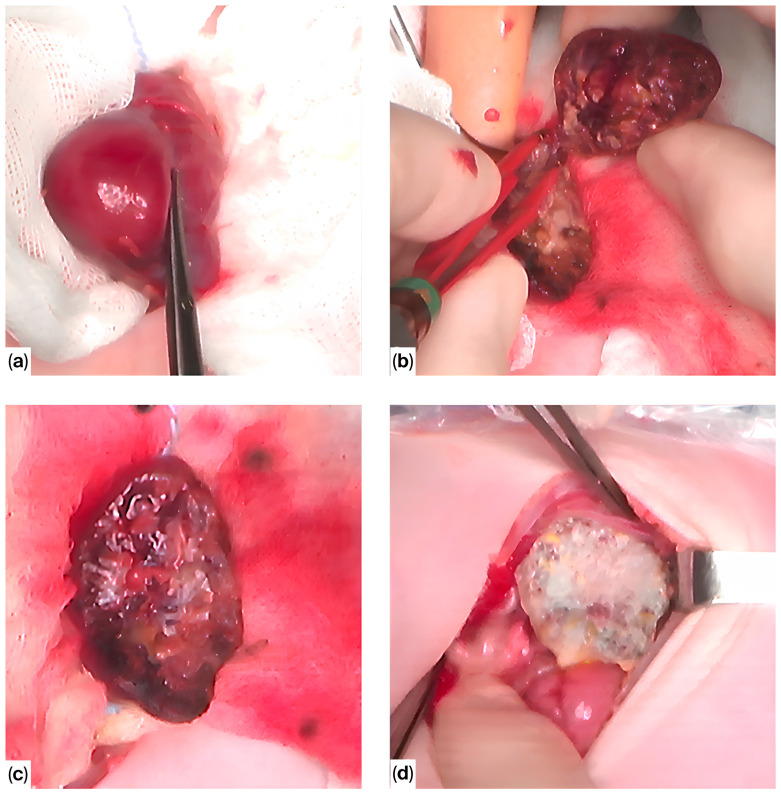
Intraoperative details. (**a**) Close-up view of the tumor along the left kidney, which was partially exteriorized to optimize surgical exposure. The surgical field was protected with gauze packing before resection. (**b**) Tumor resection with an adequate surgical margin, nearly completed, performed using bipolar forceps alone. (**c**) Appearance of the tumor bed after resection. (**d**) The parenchymal surface of the tumor bed covered with a TachoSil^®^ sponge, providing effective control of mild to moderate bleeding [9].

A full-term, male neonate weighing 2960 g was born to a 25-year-old Gravida 5 Para 4 woman, following an uncomplicated pregnancy and delivery. A second-trimester ultrasound examination had been reported as normal. Physical examination at birth revealed an apparently healthy neonate with a small, hard, palpable mass in the left upper quadrant of the abdomen. He had normal male external genitalia and no dysmorphic features or phenotypic findings suggestive of a genetic syndrome. Laboratory investigations demonstrated normal renal function and normal urinary catecholamine metabolite levels. Urinalysis was unremarkable, and systemic blood pressure was within normal limits. Abdominal ultrasonography revealed a 21 mm × 21 mm × 16 mm, isoechoic, oval-shaped, solid mass located in the lower part of the left kidney, extending cranially in close proximity to the collecting system (Figure 1). Abdominal magnetic resonance imaging (MRI) confirmed a 22 mm × 20 mm × 18 mm, homogeneous lesion lying in the lower pole of the left kidney (Figure 2). No evidence of extrarenal extension, vascular involvement, or lymph node involvement was identified. The suitability and feasibility of NSS were assessed at a multidisciplinary team meeting, and after parental consent, surgery was performed on day 9 of life via an open transperitoneal approach. The left kidney was partially mobilized to allow exteriorization of the lower pole and midportion. Intraoperatively, NSS was deemed technically feasible. Renal parenchymal transection was readily accomplished using bipolar cautery forceps alone, and the tumor was excised en bloc with a 5 mm rim of healthy renal parenchyma (Figure 3). Frozen-section analysis of the tumor bed confirmed negative surgical margins. Hemostasis was easily achieved by gentle manual compression of renal parenchyma. Following coverage of the raw surface with a TachoSil^®^ sponge, the kidney was repositioned back to the renal fossa [9]. Para-aortic lymph node sampling was performed prior to abdominal wall closure. The patient tolerated the procedure well, with negligible blood loss and no perioperative complications. Histopathological examination revealed a WT, classified as Stage II due to renal sinus invasion, and categorized as intermediate risk according to the UMBRELLA SIOP-RTSG 2016 protocol [10]. Postoperatively, the patient received 26 weeks of vincristine monotherapy, as recommended for patients weighing less than 5 kg.

At 1 year after completion of therapy, the patient remains well and disease-free. The estimated glomerular filtration rate (eGFR), calculated using the updated bedside Schwartz equation (patients ≤ 17 years old), was 114 mL/min/1.73 m^2^ [11]. MRI demonstrated symmetric differential renal volumes, indirectly indicating preserved bilateral renal function (Figure 2).

## 3. Discussion

In neonates, congenital mesoblastic nephroma is the most prevalent renal tumor, followed by WT. Both entities are associated with an excellent prognosis, given their relatively low malignant potential in this age group. The differential diagnosis between the two tumors can be challenging because their clinical and radiologic features often overlap. However, the standard of care for neonatal renal tumors is primary radical nephrectomy with further treatment guided by tumor histology and biological behavior [1,2].

In our patient, the definitive diagnosis was established only after surgical excision and histopathological evaluation. The option of NSS was discussed at a multidisciplinary team meeting based on the apparent feasibility demonstrated by preoperative imaging, which was subsequently confirmed intraoperatively. Concerns regarding the routine use of NSS for WT include a potentially higher risk of incomplete tumor resection, intraoperative tumor rupture, and local recurrence. Additionally, given that pediatric renal tumors are typically large at presentation, only a minority of cases may be amenable to NSS. Further reluctance to adopt a broader NSS approach arises from the perception that radical nephrectomy is well tolerated with minimal long-term morbidity in children. Nonetheless, a recent report from the Childhood Cancer Survivor Study (CCSS) on long-term morbidity and mortality among survivors of unilateral, non-syndromic WT diagnosed over three decades demonstrated a 35-year cumulative incidence of late kidney failure of 2.4%, representing an approximately tenfold higher risk compared with sibling controls [13]. Notably, this occurrence was not associated with specific chemotherapy or radiotherapy exposures, suggesting that nephrectomy per se may represent an independent contributing risk factor. Accordingly, the CCSS steering committee advocated for appropriate counseling of WT survivors regarding their progressively increasing lifetime risk of kidney failure, as well as the importance of early detection and management of comorbid conditions, such as hypertension and diabetes, which may further exacerbate renal risk. Finally, they concluded that an expanded role for NSS in unilateral, non-syndromic WT is desirable and should be investigated in rigorously designed prospective trials to assess whether it can reduce long-term renal morbidity without compromising oncologic outcomes [13].

In patients with unilateral WT in the setting of predisposition syndromes, evidence from both sides of the Atlantic indicates that preoperative chemotherapy significantly reduces tumor burden [14,15]. Consequently, even well-established treatment protocols that have historically favored upfront surgery have adopted preoperative chemotherapy for this subset of patients to enhance the feasibility of NSS [15]. Additionally, in patients with WT arising in horseshoe kidneys, a large series from the National Wilms Tumor Study Group demonstrated a significantly higher risk of nephrectomy among those undergoing primary resection compared with those receiving neoadjuvant chemotherapy [16]. Notably, the UMBRELLA SIOP-RTSG 2016 treatment protocol, which is one of the largest worldwide protocols incorporating chemotherapy as first-line therapy for unilateral renal tumors, has introduced a unique, dedicated section outlining stringent criteria for the application of NSS in unilateral, non-syndromic WT, rather than leaving the decision entirely to surgeon discretion [10]. Proponents of this protocol also consider preoperative chemotherapy a necessary prerequisite to maximize the feasibility of NSS [3].

Therefore, despite the aforementioned concerns, NSS is a safe and effective option for selected unilateral, non-syndromic WT when complete resection with negative margins is achievable in experienced hands. Over the past 3 decades, we have developed and refined an institutional protocol seeking to investigate the feasibility and efficacy of NSS in unilateral renal tumors, including non-syndromic WT [17]. Our experience, although limited, suggests that in carefully selected cases, NSS provides oncologic outcomes comparable to radical nephrectomy, while offering a clear advantage in preserving renal function [18]. We have also documented a strong positive correlation between split renal function quantified by DMSA scintigraphy and renal parenchymal volume of both the operated and contralateral kidneys, as estimated using ultrasonography using the ellipsoid formula [11]. A comparable correlation between ultrasonography- and MRI-based renal volumetry has been previously reported, supporting ultrasonography as a reliable and widely accessible alternative for renal volume assessment, particularly when MRI is not available [12]. Therefore, renal parenchymal volume, given its direct correlation with functional renal contribution, may serve as a surrogate marker of split renal function and a predictor of functional recovery after NSS (Figure 2).

To our knowledge, the present case represents the youngest patient and only the second reported neonatal case of unilateral, non-syndromic WT managed with NSS. The previously reported patient underwent primary NSS at 28 days of life followed by adjuvant chemotherapy, with follow-up limited to 5 months [8]. We also performed an extensive narrative review of the literature on neonates with WT treated with NSS to summarize the available evidence (Table 1).

## 4. Conclusions

The present case supports consideration of NSS in neonatal unilateral renal tumors when technically feasible and when strict oncologic principles are maintained. Neonates may derive particular benefit from NSS given the excellent long-term survival associated with contemporary WT management. Careful patient selection, meticulous surgical technique, and multidisciplinary decision-making remain essential to ensure safe and durable outcomes.

Although radical nephrectomy remains the standard of care for most unilateral WTs, preservation of renal function without compromising oncologic safety is an emerging priority. While high-level evidence is needed to support broader adoption of nephron-sparing approaches in children with unilateral WT, well-documented individual cases such as this may provide valuable insights to inform future practice and expand management paradigms.

## Figures and Tables

**Table 1:** Characteristics, treatment, and outcomes of reported neonatal Wilms tumors managed with nephron-sparing surgery to date.

Study	Sex	Age at Surgery	Stage	Histology	Initial Treatment	Further Treatment, Outcome (Follow-Up)
Cecchetto 1990 [5]	male	20 days	V	WT	Pre-op CT (VCR) Right NSS and left nephrectomy	Post-op CT (NA), RT, Second-look surgery, Exitus
Gordon 1996 [7]	female	NA	V	WT	Bilateral NSS	Post-op CT (AV), Negative second-look surgery (8 months)
Streif 1997 [4]	male	NA	I	CPDN	Left NSS	Disease-free (5 years)
Meng 2021 [6]	NA	3 months	V	WT	Pre-op CT (AV), left NSS, 4-drug CT, right NSS	4-drug post-op CT, Disease-free (1 year)
Sarin 2024 [8]	male	28 days	I	WT	Left NSS	Post-op CT (AV), Disease-free (5 months)
Ceccanti 2026 (This study)	male	9 days	II	WT	Left NSS	Post-op CT (VCR) Disease-free (18 months)

Abbreviations: AV—actinomycin/vincristine; CPDN—cystic partially differentiated nephroblastoma; CT—chemotherapy; NA—not available; NSS—nephron-sparing surgery; RT—radiotherapy; VCR—vincristine; WT—Wilms tumor.

## Data Availability

All data generated or analyzed during this study are included in this article. Further enquiries can be directed to the corresponding author upon reasonable request.
